# Resignation of Prof. Dudley

**Published:** 1850-08

**Authors:** 


RESIGNATION OF PROFESSOR DUDLEY.

We learn, from the *Transylvania Journal of Medicine*, that Prof. B. W. Dudley has resigned the chair of Surgery, which he has occupied for more than a quarter of a century. Dr. Dudley is well known as one of the most distinguished surgeons of our country, and is especially celebrated for his unprecedented success in the operation of lithotomy.

